# New Drugs. Appliances, and Things Medical

**Published:** 1893-04-15

**Authors:** 


### NEW DRUGS, APPLIANCES, AND THINGS MEDICAL.

[All preparations, appliances, novelties, etc., of which a notice is desired, should be sent for the Editor, to care of The Manager, 428, Strand, London, W.C.]

### OSWEGO PREPARED CORN.

(Kingsford and Co., 44, Upper Thames Street.)

Oswego cornflour has long held its own in the market on account of the excellence of its quality. Cornflours are amongst the commodities which vary little in kind. Of the Oswego cornflour it can fairly be said that the "starchy flavour" peculiar to cornflours is more easily overcome in cooking than in most kinds we know. It is especially suitable, therefore, in making custards and blanc manges, and lends itself to a variety of combinations alike suitable for household use and the sick-room. It is manufactured from the finest part of Indian corn, and is free from adulteration.

### THE MAGIC CYCLE AND GENERAL OILER.

(Oscar Moenich and Co., 8, Coleman Street, E.C.)

This new American oiler is a most admirable little contrivance in every respect. Cycling is so popular and healthy a mode of exercise that much attention is paid by inventors to the supply of every convenience. We have seen no oiler more convenient for the traveller's use than that before us. Whilst the receptacle is ample, the oiler measures but two and a-half inches when closed. A cap securely closes the oiler with a spring. The flow is under the users regulation, it does not leak, and is most perfectly cleanly both in itself and in the manner of use. While especially commending itself to cyclists, it will be found of use whereever machinery necessitates the use of an oiler. We predict that it will become most popular when known. The cost is 1s. 6d.

### MILK FOOD FOR INFANTS.

(Henri Nestle, 9, Snow Hill, E.C.)

To find a really wholesome and nutritious food for infants is one of the greatest difficulties of the nursery. Great improvements have certainly been made of late years in this direction. The poor who, above all, require to be able to procure suitable diet for their infants especially need education on this point. The use of Nestle's milk food (*Lacteous Farina*), could the public be made to appreciate its value, would save the life of many a child doomed to premature death through injudicious diet alone. Nestle's food is alike valuable to rich and poor, containing as it does all the constituents necessary to form a diet for children in infancy as a substitute for milk during periods when this cannot be retained by the child, and also to supplement a milk diet. It consists of wheat, sugar, and milk of excellent quality, and is carefully prepared to closely resemble mothers' milk. A long record of successful use bears testimony to the excellency of the preparation, which besides being so useful in the nursery, is an excellent food for invalids also, being easy of digestion and pleasant to the taste.

### IMPROVED CLINICAL IMPERISHABLE INDEX THERMOMETERS.

(F. Darton and Co., Clerkenwell Optical Works, 142 St. John Street, E.C.)

This firm supply clinical thermometers of good quality at a wonderfully low price. Considering the extremely perishable nature of these articles of daily use in hospitals, cost is a very important consideration. Messrs. Darton offer their hospital clinical thermometers at 18s. per dozen in metal cases. These are serviceable and reliable little thermometers, and quite adapted to ordinary use. The improved imperishable index thermometers are slightly more expensive, and can be supplied six instead of four inches in length. The "Rapid Action" are capable of registering at thirty seconds, and even these are but 27s. per dozen. We specially recommend the thermometers with magnifying lens front, and all varieties with flattened side; this flattening obviates the tendency to roll, which is often responsible for breakage. Centigrade scale thermometers are supplied at the same price, and the Kew certificate can be had for an extra payment. Messrs. Dartons' thermometers are worth a trial, and will prove, we feel sure, universally acceptable.

### TRITICUMINA FOOD.

(Messrs. Meaby, Reading, and 7, St. Paul's Churchyard, E.C.)

Messrs. Meaby's Triticumina Food is a wholesome, nourishing, and agreeable product. As a food for children it is excellent, and will be popular with all who partake of foods of this description, as it has a flavour peculiarly its own, rendering it less insipid than many others of its kind. The starch of the grain used in its composition is converted into dextrine; it is rich in mineral matter, and the fine portions of the inner bran are retained. Prepared with milk this food will prove a most beneficial and welcome addition to the diet of sick persons and children.

